# Correlations between Immune Response and Etiopathogenic Factors of Medication-Related Osteonecrosis of the Jaw in Cancer Patients Treated with Zoledronic Acid

**DOI:** 10.3390/ijms241814345

**Published:** 2023-09-20

**Authors:** George Adrian Ciobanu, Laurențiu Mogoantă, Sanda Mihaela Popescu, Mihaela Ionescu, Cristina Maria Munteanu, Ionela Elisabeta Staicu, Răzvan Mercuț, Cristian Corneliu Georgescu, Monica Scrieciu, Daniel Vlad, Adrian Camen

**Affiliations:** 1 Department of Oral Rehabilitation, University of Medicine and Pharmacy of Craiova, 200349 Craiova, Romania; 2 Department of Oral and Maxillofacial Surgery, Dental Medicine Faculty, Ovidius University of Constanța, 900470 Constanța, Romania; 3 Department of Histology, University of Medicine and Pharmacy of Craiova, 200349 Craiova, Romania; 4 Department of Medical Informatics and Biostatistics, University of Medicine and Pharmacy of Craiova, 200349 Craiova, Romania; 5 Department of Oral and Maxillofacial Surgery, University of Medicine and Pharmacy of Craiova, 200349 Craiova, Romania; 6 Department of Orthodontics, University of Medicine and Pharmacy of Craiova, 200349 Craiova, Romania; 7 Department of Plastic Surgery, University of Medicine and Pharmacy of Craiova, 200349 Craiova, Romania; 8 Department of Pharmacology, University of Medicine and Pharmacy of Craiova, 200349 Craiova, Romania; 9 Department of Prosthetic Dentistry, University of Medicine and Pharmacy of Craiova, 200349 Craiova, Romania

**Keywords:** MRONJ, zoledronic acid, immunohistochemical markers

## Abstract

Impairment of the immune response in MRONJ (medication-related osteonecrosis of the jaws) is one of the still unclear etiopathogenic mechanisms of this condition encountered in cancer patients treated with bisphosphonates, with negative effects on the patient’s quality of life. The aim of the present study was to correlate the immune response with etiopathogenic factors via immunohistochemical evaluation of the maxillary tissues in zoledronic acid osteonecrosis. The retrospective study included a group of 51 patients with various types of cancers, diagnosed with stage 2 or 3 MRONJ at zoledronic acid and treated surgically. Immunohistochemical expressions of αSMA, CD3, CD4, CD8, CD20, CD79α, CD68, CD204, and tryptase were evaluated. Immunohistochemical markers expressions were statistically analyzed according to the duration of the treatment, the trigger factor, the location of the MRONJ, and the healing status. Analysis of the immune response included T lymphocytes, B lymphocytes, plasma cells, macrophages, and mast cells. The duration of treatment significantly influenced the immunohistochemical expression of most markers (*p* < 0.05). For an increasing trend in treatment duration, a decreasing trend in marker score was observed, suggesting an inverse correlation. The expression of the markers was different depending on the trigger factor, on MRONJ localization (maxilla/mandible), and the healing status, being more intense in patients cured per primam compared to those who had relapses. The patient’s immune response was negatively influenced by the duration of the treatment, the trigger factor, the location of the lesion in the mandible, and the recurrence of MRONJ.

## 1. Introduction

Osteonecrosis of the jaw occurring as an adverse effect of antiresorptive and antiangiogenic medication, is a multifactorial condition that greatly reduces the quality of life of cancer patients [1,2,3,4,5]. Originally defined as bisphosphonate-induced osteonecrosis of the jaws (or BRONJ) [6], it was renamed in 2014 to medication-related osteonecrosis of the jaws (or MRONJ), since, in addition to bisphosphonates (BPs), other antiangiogenic and antiresorptive drugs with osteonecrosis-like adverse effects are involved [1,7]. Among the bisphosphonates, zoledronic acid is the most potent drug, but it also has the strongest adverse effects such as osteonecrosis in the jaw bones [1,8,9,10]. It is used as the first treatment option for bone metastases in patients with various types of cancer, of which breast, ovarian, or prostate adenoma are the most common. The treatment regimen with zoledronic acid involves its intravenous administration at a dose of 4 mg monthly, accompanied by dexamethasone [11,12]. The frequency of bisphosphonates osteonecrosis in cancer patients with bone metastases varies between 0.5% and 18%, depending on the studied group [1,9,10,13,14]. Among the risk factors involved in its occurrence, in addition to drug-related factors (potency, route of administration, duration of treatment, pharmacokinetic and pharmacodynamic characteristics, and drug combinations) [4,15,16], there are patient-related factors, namely genetic factors [17], systemic factors such as the presence of coexisting diseases, such as diabetes mellitus (DM) [18], hypertension (HTN) [4]), or the development of bone metastases [19], and oral local factors. Oral factors include the presence of foci of intra-osseous infection such as chronic apical periodontitis or advanced chronic periodontitis, vertical root fractures, mucosal or periodontal trauma produced by prosthetic work, all aggravated by poor oral hygiene, as well as maxillary surgical interventions that do not achieve primary closure of the wound, such as extractions [4,16,20]. The mechanism of MRONJ is not yet fully understood [21]. MRONJ occurs exclusively in the jaw bones covered by the oral mucosa, an oral barrier provided with one of the most active barrier immunities [22]. As a result of their pharmacokinetics, BPs are deposited in the jawbones, their half-life exceeding 10 years [23]. Bisphosphonates in jawbones interact uniquely with oral barrier immunity. Okawa et al. [24] showed that in MRONJ, there is an immune dysregulation of the oral barrier and a pro-inflammatory reaction. In oral tissues, BPs have negative effects on vascularization, on the bone and the oral epithelium. Thus, in experimental studies on animals, it appears a reduction in the number of bone cells, osteoclasts, osteoblasts, and osteocytes [25,26], a reduction in the number of new vessels in oral wounds [25], and keratinocyte apoptosis with impairment of wound healing [27,28]. To elucidate the mechanism of MRONJ occurrence, Okawa et al. [24] performed a study on mice by selectively replacing zoledronic acid in the jaw bones with hydroxy methylene diphosphonate (HMDP) applied intraorally. Topical applications to BRONJ lesions of a lower-potency bisphosphonate in the form of a liposome-based nanoscale deformable vesicle product, resulted in accelerated gingival wound closure and bony socket healing, as well as attenuation of osteonecrosis development. At the same time, a resolution of chronic inflammation was observed by increasing the gene expression of the anti-inflammatory signature of lymphocytes and myeloid-derived suppressor cells [24]. Several theories were proposed for the pathogenesis of MRONJ [21], suggesting a multifactorial origin [16,21,29,30,31,32]. Since a lot of studies that provide evidence regarding etiopathogenic mechanisms were carried out mainly in animal models, the use of immunohistochemical analysis performed on collected tissue from patients with MRONJ from surgically treated oral wounds could bring new information related to the etiopathogenic mechanisms of MRONJ. From other research carried out on MRONJ [33,34,35], the pathophysiology of MRONJ seems to be multifactorial, with the infection, inflammation, and trauma of the oral tissues amplified by the alteration of bone remodeling or excessive suppression of bone resorption and inhibition of angiogenesis [16,29,30,31]. Besides these factors, the etiopathogenic mechanisms of osteonecrosis include toxic effects on keratinocytes and the impairment of the immune response via the effects on immune cells (T-lymphocytes, B-lymphocytes, plasma cells, macrophages, and mast cells).

The objective of this retrospective study was to correlate the immune response defined by the expression of immunohistochemical markers (αSMA, CD3, CD4, CD8, CD20, CD79α, CD68, CD204, and tryptase) with etiopathogenic factors of MRONJ by using the information obtained from the immunohistochemical analysis of MRONJ tissues from cancer patients treated with zoledronic acid.

## 2. Results

### 2.1. Epidemiological Aspects in Study Patients

The study group included 51 patients, 26 from Craiova and 25 from Constanta, with ages between 46 and 85 years old, mean value of 69.94 ± 8.50 years old, comprising 32 females (representing 62.7% of the entire study lot) and 19 males (37.3%).

**Figure 1:**
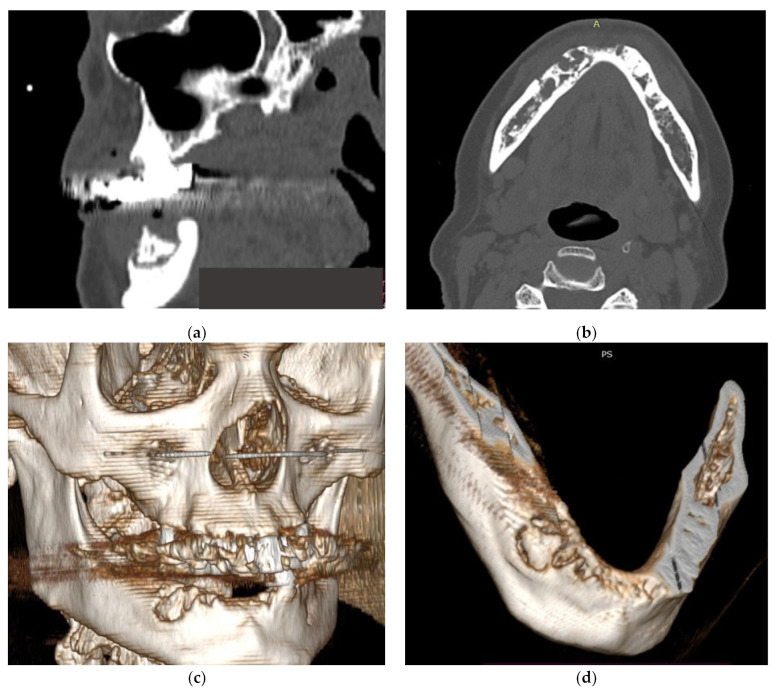
(**a**–**d**) CT aspects of patients with MRONJ.

**Figure 2:**
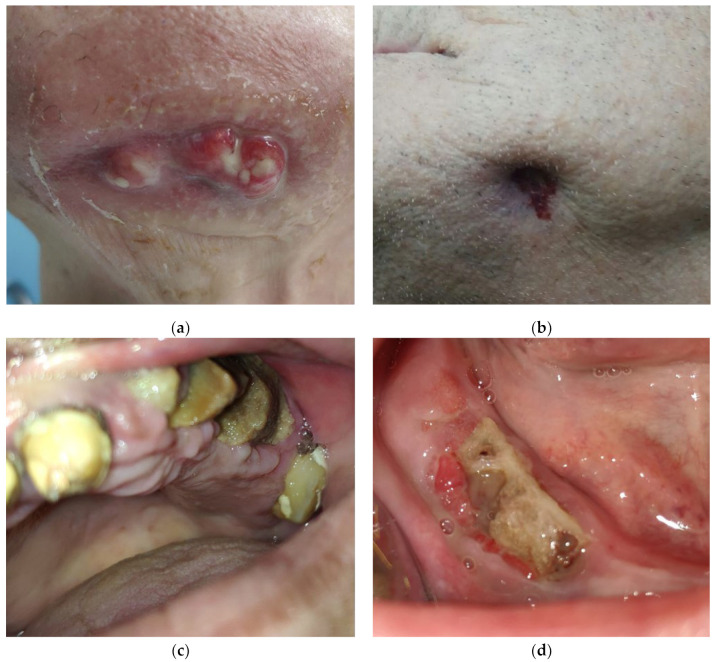
(**a**–**d**) Clinical aspects of patients with MRONJ.

All patients were diagnosed with cancer and bone metastasis and developed MRONJ (Figure 1). Patients reported local radiating pain in both jaws, with oral dysfunction (mostly with an altered masticatory function or physiognomic alterations for patients with MRONJ in the frontal area of the jaws) (Figure 2). One patient complained of paresthesia in the lower jaw.

The most frequent location of the primary tumor was the breast (49.02%), followed by the prostate (29.41%) and colon (7.84%). Most of the patients in the study lot suffered from comorbidities such as hypertension (32 patients, 62.75%) or obesity (14 patients, 27.45%).

All patients were treated with zoledronic acid (ZA), administered intravenously at 4 mg once a month, and osteonecrosis appeared after a period varying between 24 and 35 months (for 30 patients, 58.8%), and between 36 and 48 months (for 21 patients, 41.2%). BP treatment duration varied between 12 and 48 months, with a mean value of 29.51 ± 9.50 months (approx. 2.5 years).

The trigger factor for MRONJ development was extraction (for 34 patients, 66.7%), periapical infection (for 10 patients, 19.6%), and periodontal disease (for 7 patients, 13.7%), and in most cases, the local pain appeared spontaneously. MRONJ location was mostly at the lower jaw for 34 patients (66.7%), compared to only 17 patients with MRONJ at the upper jaw (33.3%).

Most patients diagnosed and treated for osteonecrosis presented stage 2 MRONJ (39 patients, representing 77.6% from the entire study lot), and the rest of the 12 patients (22.4%) had stage 3 MRONJ. Almost all patients had a poor health status, with ulcerations and exposure of the necrotic bone in 64.7% of cases, fistulae (41.17%), or even abscesses and purulent secretions (29.41%). Two patients with stage 3 upper jaw MRONJ presented maxillary sinusitis. All patients were surgically treated, and samples of tissues were analyzed histopathologically and immunohistochemically.

Relapse occurred for 13 patients (25.5%), while 38 patients (74.5%) were considered healed after the first surgical intervention. There was a statistically significant association between relapse status and MRONJ stage, χ^2^(1) = 11.754, *p* = 0.002 (Fisher’s Exact test). The association was strong with Cramer’s V = 0.490, as more than half of the patients with MRONJ stage 3 suffered a relapse after the first surgical intervention (63.6%), compared to patients with MRONJ stage 2, where only 13.2% experienced relapse (Table 1).

A point-biserial correlation was run between relapse status and age. Preliminary analyses showed there were no outliers, as assessed by boxplot; ages were normally distributed (*p* > 0.05), and variances were homogeneous. There was a statistically significant correlation between relapse status and age, r_pb_(49) = 0.314, *p* = 0.025, with relapse occurring more in older patients than in younger patients (74.461 ± 8.79 versus 68.394 ± 7.93) (Table 1). The same test was run between relapse status and BP treatment duration. There was no statistically significant correlation between relapse status and treatment duration, r_pb_(49) = 0.227, *p* = 0.110, even if relapse occurred more for patients with an increased treatment duration rather than a smaller treatment duration (33.153 ± 10.78 versus 28.263 ± 8.82) (Table 1).

Bacterial colonies, inflammatory infiltrate, and lymphoplasmacytic infiltrate were present in more than half of all patients, as indicated in Table 2, with around 75% of them representing patients who were healed after the first surgical intervention. Viable bone was present for only 31.4% of the entire study lot (16 patients), mostly among healed patients, but with no statistically significant association with the relapse status (Table 2).

### 2.2. Histopathological Aspects of MRONJ in Bisphosphonates in Cancer Patients

**Figure 3:**
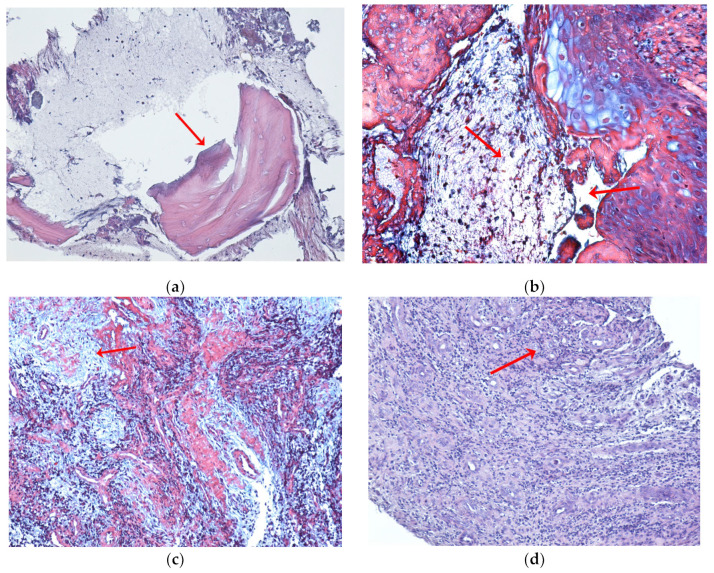
Histopathological aspects of bone and marginal periodontium in MRONJ to zoledronic acid in cancer patients (red arrows). (**a**) Maxillary bone with an area of necrosis with a moth-eaten appearance and inflamed periodontium. HE, ×100. (**b**) Necrotic bone and connective tissue with the presence of cell nuclei in the area of necrosis. ×100, trichrome stain. (**c**) Diffuse inflammatory infiltrate that alters (dissects) the structure; abundant, chronic lympho-plasmacytic inflammatory infiltrate, numerous blood capillaries with turgescent endothelium; HE, ×100. (**d**) Periodontium with diffuse chronic inflammatory infiltrate, multinucleated giant cells arising from the union of macrophages as a reaction to a foreign body, with numerous newly formed capillaries. HE, ×100.

Various histopathological aspects of the bone tissue were highlighted in the examined samples. These ranged from areas of bone necrosis that alternated with areas of bone tissue in different stages of degradation, intact bone tissue, and bone tissue with early osteonecrosis to bone tissue that was completely necrotic, resulting in a mosaic appearance. Areas of advanced osteonecrosis showed an inhomogeneous, vacuolar appearance with irregular borders and a “moth-eaten” appearance, empty osteocytic lacunae with no surrounding defense reaction, no osteoblasts, and no osteoclasts, and Haversian canals with central bone necrosis (Figure 3a,b). In the examined fragments, the deep marginal periodontium around the area of necrosis showed, as a defense reaction of the body, an abundant inflammatory infiltrate with neutrophil cells, lymphocytes, and macrophages (Figure 3c,d).

**Figure 4:**
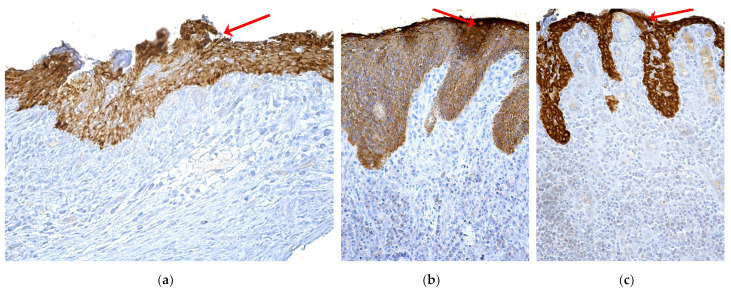
Immunohistochemical aspects of the marginal periodontium in MRONJ (red arrows). (**a**) Periodontium with necrotic and denuded superficial epithelium and chorion infiltrated with immune cells; ×200, AE1/AE3. (**b**) Periodontal fragment with intensely positive epithelial AE1/AE3 immunolabeling, with underlying chorion with abundant inflammatory infiltrate. ×200, AE1/AE3. (**c**) Periodontal fragment with thin epithelium intense positive AE1/AE3 immunolabeling, and chorion with rich inflammatory and hemorrhagic infiltrate; ×200, AE1-AE3.

Immunohistochemical images of oral mucosa fragments showed damage to the oral epithelium and the chorion. In epithelium, a positive AE1/AE3 immunohistochemical reaction was revealed, which varied from moderately positive in areas with epithelial necrosis and disruption of the superficial layer to an intensely positive reaction in areas with thickened or spongy epithelium and the presence of intraepithelial inflammatory cells. The underlying chorion was characterized by the presence of moderate infiltrate with immune cells in fragments with epithelial necrosis and by the presence of abundant diffuse inflammatory infiltrate in fragments with thickened or spongy epithelium (Figure 4a–c).

### 2.3. Results of MRONJ Immunohistochemical Analysis on Bisphosphonates in Patients with Cancer—Expression of IHC Markers in the Studied Group

Markers’ expressions were evaluated as Negative (N), Low (L), Moderate (M), and Intense (I). Table 3 reflects the markers’ expression for immunohistochemical analysis carried out on tissue fragments harvested from MRONJ patients.

#### 2.3.1. IHC Markers’ Expression for the Study Group, Divided by the Two Geographical Regions

A Mann–Whitney U test was run to determine if there were differences in markers’ expression between patients enrolled in the two medical centers. Distributions of the expression levels for the two groups of patients were not similar, as assessed by visual inspection. The expressions for the patients in Craiova were overall higher for all markers compared to patients from Constanta, and the differences were not statistically significant *p* > 0.05, for 7 out of 9 markers. For two markers, the expressions were statistically significantly different between the two medical centers: for αSMA: U = 213, z = −2.379, *p* = 0.017, and for MASTOCYTES, with U = 220, z = −2.102, *p* = 0.036.

#### 2.3.2. IHC Markers’ Expression for the Study Group, Divided by the Duration of BP Treatment

Kendall’s tau-b correlation was run to determine the relationship between BP treatment duration and IHC markers’ expression amongst the 51 patients included in the study group. For 8 of 9 markers (all except CD79), there was a moderate or strong negative association between treatment duration and markers’ expression, which was statistically significant, *p* < 0.05 (Table 4).

**Figure 5:**
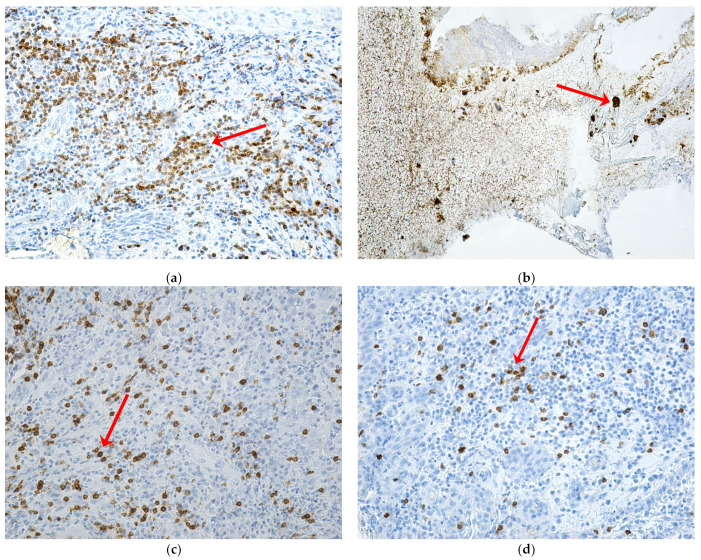
Expression of CD3, CD4, and CD8 markers in patients with MRONJ (red arrows): (**a**) periodontium with T lymphocytes, with intensely positive CD3 immunolabeling, and with focal distribution, ×200; (**b**) mucosal and bone fragment with T lymphocytes, with CD4 immunomarking absent in the focus of bone necrosis but intensely positive in adjacent areas ×200; (**c**) periodontium with rare T lymphocytes, with intense positive CD8 immunolabeling, and with in-homogeneous distribution, ×200; (**d**) periodontium with rare T lymphocytes, with intensely positive CD8 immunolabeling in areas with inflammatory infiltrate and absent in areas of neoangiogenesis, ×200.

T lymphocytes were present in low numbers in tissues affected by osteonecrosis with weakly positive immunolabeling. Immunoexpression of CD3, CD4, and CD8 markers was moderate intense for images from the periodontium (Figure 5).

**Figure 6:**
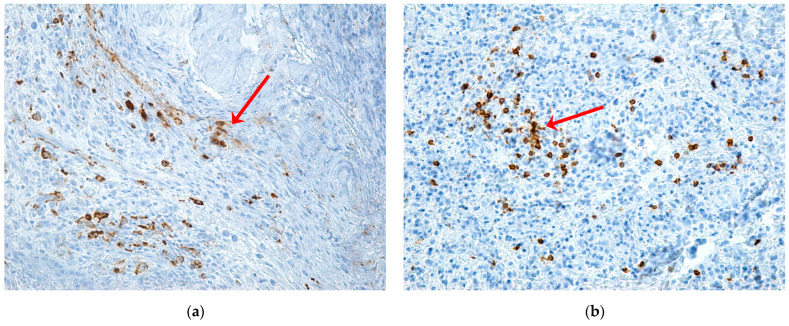

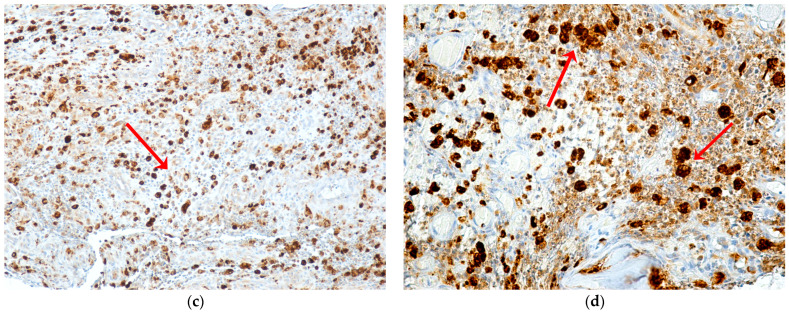
Expression of CD68, CD204 markers for macrophages in patients with MRONJ (red arrows): (**a**) muco-osseous fragment with rare macrophages, with moderately positive CD204 immunoexpression, mainly distributed in the areas with inflammatory infiltrate and adjacent to the area of osteonecrosis, ×20; (**b**) periodontium with rare macrophages, with intensely positive CD68 immunomarking in the inflammatory focus, ×20; (**c**) periodontium with numerous macrophages with intensely positive CD68 immunoexpression, in the areas with rich inflammatory infiltrate, ×100; (**d**) periodontium with numerous macrophages of predominantly large sizes, with intensely positive CD68 immunolabeling, ×200.

Macrophages were observed in the periodontium in large numbers in the areas with rich inflammatory infiltrate, showing intensely positive CD68 expression. They were rare in the muco-osseous fragments with necrosis, showing moderate positive immunolabeling (Figure 6).

**Figure 7:**
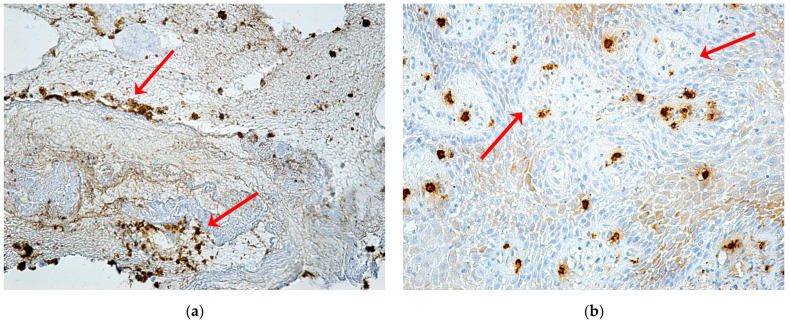

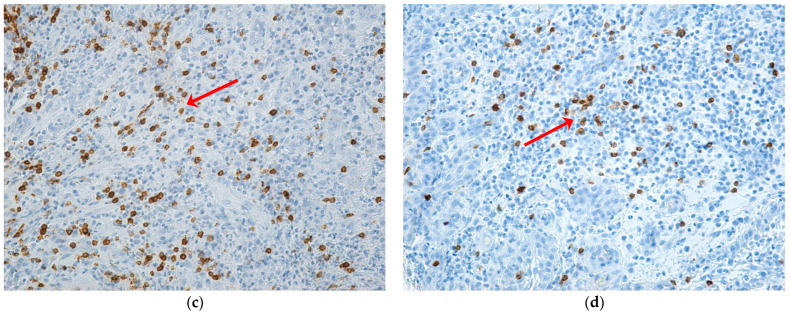
Expression of the tryptase marker (red arrows): (**a**) bone fragment with a mosaic appearance, with areas of necrosis in different evolutionary phases and with numerous intensely positive mast cells at the periphery of the osteonecrosis areas, ×20; (**b**) gingival epithelium with numerous connective papillae and intraepithelial mast cells, with moderate and weakly positive immunolabeling and mast cells in the chorion with intense positive immunolabeling, ×200; (**c**) periodontium with numerous intensely positive mast cells and with diffuse distribution, ×200; (**d**) periodontium with inflammatory infiltrate and clustered intensely positive mast cells, ×200.

Mast cells were observed in both bone and periodontal tissue fragments. In the bone tissue, mast cells immunolabeled for tryptase were identified in large numbers at the periphery of the osteonecrosis zone and the periodontal tissue, both intraepithelial and in the underlying chorion (Figure 7).

#### Markers’ Score

**Figure 8:**
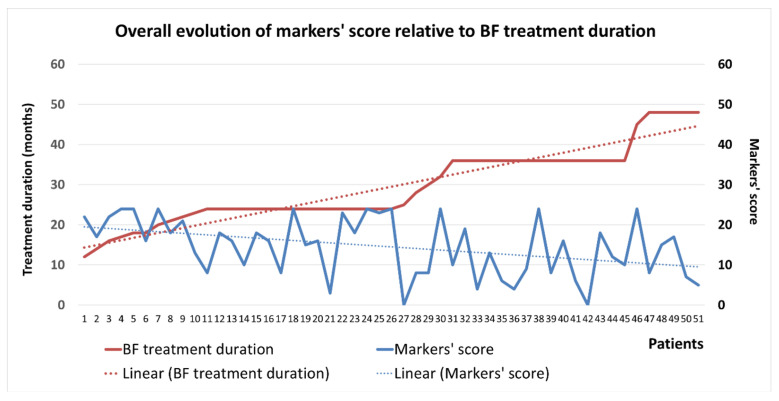
Evolution of IHC markers’ score in relation to BP treatment duration.

Each possible value of markers was numerically quantified, to obtain a general score per patient (Negative = 0, Low = 1, Moderate = 2, Intense = 3). This score was the sum of all markers’ values, thus obtaining one single numerical value for every patient included in the study lot. Figure 8 presents the generic evolution of this score related to the BP treatment duration, showing that for an increased treatment duration trend, we obtain in opposition a decreased trend of the markers’ score. The average markers’ score at the beginning of the BP treatment is around 20 and decreases (until around 10) while the BP treatment period increases.

#### 2.3.3. IHC Markers’ Expression for the Study Group, Divided by the Trigger Factor

A Kruskal–Wallis test was conducted to determine if there were differences in markers’ expression between MRONJ trigger factors: the “periodontal disease” (n = 7), “extraction” (n = 34), and “periapical infection” (n = 10) trigger factors. Distributions of markers’ expressions were not similar for all groups, as assessed by visual inspection of a boxplot. Overall, the markers’ expression for MRONJ triggered by periodontal disease was higher (Moderate–Intense) for all markers, followed by lower values for MRONJ triggered by extractions (Low–Moderate), and Low values for MRONJ triggered by periapical infection. Only for αSMA, the expressions were statistically significantly different between the different trigger factors, χ^2^(2) = 6.061, *p* = 0.048. Subsequently, pairwise comparisons were performed using Dunn’s (1964) procedure with a Bonferroni correction for multiple comparisons. This post hoc analysis revealed statistically significant differences in the markers’ expression between periodontal disease (35.86) and periapical infection (19.90) (*p* = 0.042), but not between other group combinations (Table 5).

**Figure 9:**
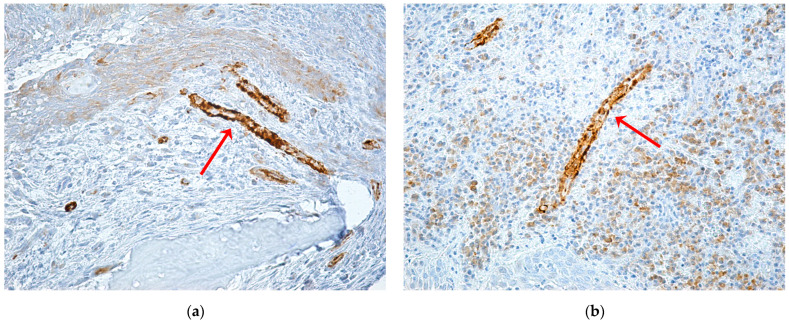

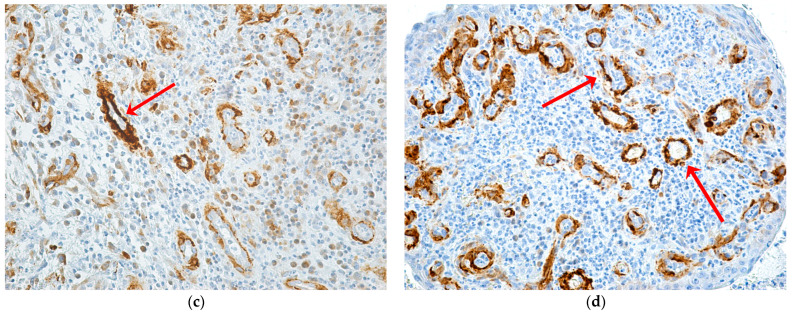
Alpha SMA expression according to the trigger factor (red arrows): (**a**) mucoperiosteal fragment with very few blood vessels, showing intensely positive αSMA immunomarking, ×200; (**b**) periodontium with abundant inflammatory infiltrate and moderately positive intratissue αSMA immunomarking with diffuse distribution and intense positive immunomarking in the vascular wall, ×200; (**c**) periodontium with moderately diffuse inflammatory infiltrate, and numerous blood vessels with moderately and intensely positive developing immunolabeling, ×200; (**d**) periodontium with abundant inflammatory infiltrate and numerous new formed vessels with intense positive immunomarking, ×200.

The intensity of the αSMA immunohistochemical reaction varied in appearance from low intensity with the significance of reduced vascularization in the areas of inflammation to high intensity with the significance of rich vascularization with numerous vessels of angiogenesis, in the case of patients with an abundant inflammatory reaction and high regeneration capacity (Figure 9).

**Figure 10:**
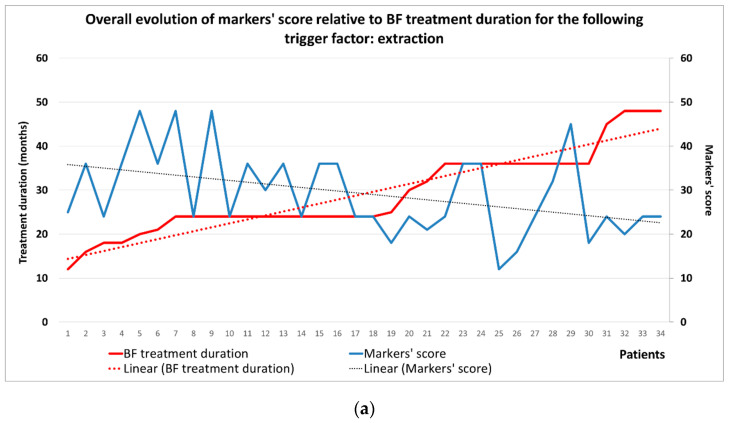

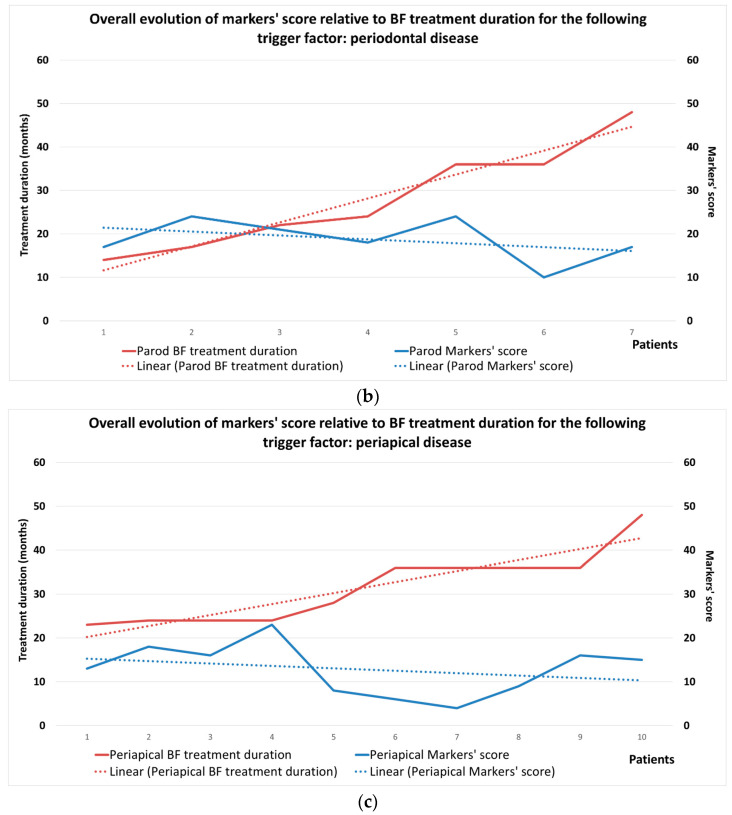
Evolution of IHC markers’ score in relation to the trigger factor and BP treatment duration: (**a**) extraction; (**b**) periodontal disease; (**c**) periapical disease.

For patients with extraction as a trigger factor, the markers’ score had the highest values at the beginning of the BP treatment (almost double compared to the initial overall average score) and maintained this characteristic even for high treatment durations, remaining around 10, similarly to the average score of the group (Figure 10a).

For patients with periodontal disease, the markers’ score at the beginning of treatment was a little higher than the average markers’ value for the entire study group and maintained this trend until the end of the study period (Figure 10b).

For patients with periapical disease, the markers’ score at the beginning was below the average for this period (Figure 10c).

#### 2.3.4. IHC Markers’ Expression for the Study Group, Divided by MRONJ Localization

Markers’ expression in the upper jaw was predominated by Intense values (Table 6). Only T/CD4 and CD204 had mostly Low or Negative expressions. For the lower jaw, the most common location of MRONJ, the Moderate expression was predominant, followed by Low (Table 6). A Mann–Whitney U test was run to determine if there were differences in markers’ expression between the upper and the lower jaw. Distributions of the expression levels for the two groups of patients were not similar, as assessed by visual inspection. The expressions for the patients with MRONJ at the upper jaw level were overall higher for all markers (mostly Intense expression) compared to the patients with MRONJ at the lower jaw level (mostly Low and Moderate expression) and the differences were not statistically significant, *p* > 0.05 for all markers.

**Figure 11:**
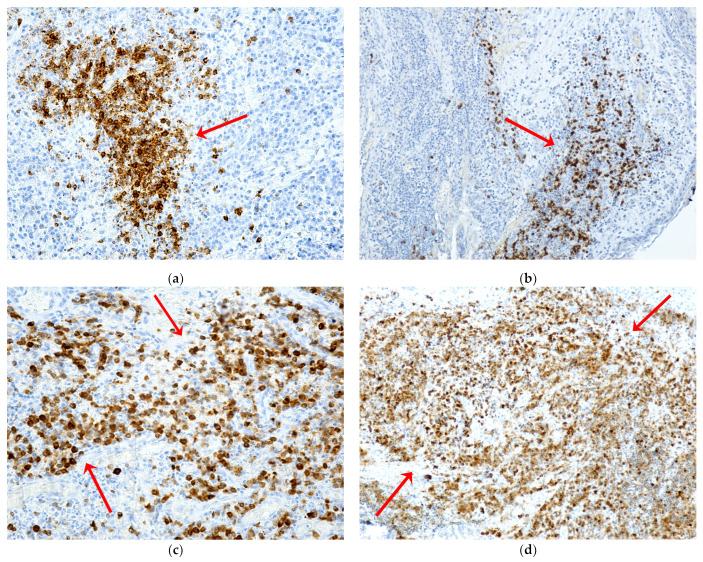
Expression of CD20 and CD79α markers in patients with MRONJ. B lymphocytes (**a**,**b**) and plasma cells (**c**,**d**) (red arrows): (**a**) periodontium with abundant inflammatory infiltrate with intensely positive immunomarked B lymphocytes with CD20, agglutinated in lymphoid follicles or disseminated ×200; (**b**) periodontium with abundant inflammatory infiltrate, with a field of B lymphocytes with intense positive CD20 immunomarking, with the inhomogeneous arrangement, ×100; (**c**) periodontium with moderate inflammatory infiltrate, with a very large number of plasma cells, with intense positive CD79α immunomarking, and with diffuse distribution, ×20; (**d**) periodontium with inflammatory infiltrate and areas of necrosis and numerous plasma cells with moderately and intensely positive CD79α immunomarking, unevenly distributed, ×10.

B lymphocytes immunolabeled with CD20 were agglutinated in lymphoid follicles or disseminated forming a field of B lymphocytes with an inhomogeneous arrangement in the inflammatory periodontium (Figure 11a,b). Plasmocytes immunolabeled with CD79α were in very large numbers in the areas of necrosis, with an intense immunohistochemical reaction (Figure 11c,d).

#### 2.3.5. IHC Markers’ Expression for the Study Group, Divided by the Relapse Status after the First Surgical Intervention

A Mann–Whitney U test was run to determine if there were differences in markers’ expression between healed and relapsed patients after surgical intervention. Distributions of the expression levels for the two groups of patients were not similar, as assessed by visual inspection. The expressions for the Healed patients were overall higher for all markers (mostly Moderate expression) compared to Relapse patients (mostly Low expression) and the differences were not statistically significant *p* > 0.05 for all markers. For the subgroup of Healed patients, the immunohistochemical analysis revealed that markers were mostly expressed as Moderate or Intense. The following markers were mostly expressed as Moderate: αSMA, T/CD8, B/CD20, PLASM/CD79, and MASTOCYTES, while Intense expression was predominant for T/CD3, T/CD4, CD68, and CD204. In contrast, the Relapse group emphasized more Low expressions. For the subgroup of Relapse patients, only αSMA and B/CD20 were mostly expressed as Moderate, while all others presented mostly Low intensities (Table 7).

A Mann–Whitney U test was also carried out to determine if there were differences in markers’ expression between gender, MRONJ stage, HTA presence, obesity, and localization. Distributions of the expression levels for each of the two groups of patients were not similar, as assessed by visual inspection. The expressions for the healed patients were overall higher for all markers (mostly moderate expression) compared to relapse patients (mostly low expression) and the differences were not statistically significant, *p* > 0.05 for all markers.

## 3. Discussion

The present study showed that zoledronic acid has negative effects on bone, periosteum, and oral epithelium, as well as on blood vessels and immune cells in oral tissues. If, at the bone level, major effects were observed that led to the death of bone cells (osteoclasts, osteocytes, and osteoblasts), and at the epithelial level the effects on keratinocytes showed destruction and ulceration of the epithelium, the expression of immunohistochemical markers varied greatly depending on the etiopathogenic factors and the prognosis of osteonecrosis healing. Patients with osteonecrosis presented with poor oral health status, the trigger factors of osteonecrosis being represented by recent extractions, periapical infections, or periodontal disease. Patients who underwent surgery as treatment for MRONJ had advanced stages of osteonecrosis, 2 and 3. More than 25% of them experienced a recurrence of the disease after the first surgery, the majority being in stage 3 osteonecrosis. Recurrence of the disease occurred especially in elderly patients. Although the longer the duration of treatment, the number of patients with relapse increased, this correlation was not statistically significant for the study group.

Despite the increasing amount of literature generated over the years (1400 articles only in the last 6 months), the pathogenesis of MRONJ is still not fully elucidated. Several theories have been proposed for the pathogenesis of MRONJ [21,36,37], including suppression of bone remodeling, inhibition of angiogenesis, infection and inflammation, soft tissue toxicity, and oxidative stress [37].

From other research on MRONJ [16,29,30,31,32,33,34,35,38], five directions of analysis of the etiopathogenic mechanisms may be outlined: the changes in the jaw bone, with the suppression of bone turnover via the dysfunction and death of bone cells, the inhibition of angiogenesis via the effects on blood vessels, the toxicity of BPs on epithelial cells, keratinocytes, the impairment of the response immune via the effects on immune cells (T lymphocytes, B lymphocytes, plasma cells, macrophages, mast cells), all of which take place in an environment where infection with various microbial species is present, from bacteria to fungi, viruses, and other types of microorganisms.

MRONJ occurs exclusively in the jawbones, associated with a very active oral tissue barrier from an immune perspective [39]. The unique characteristics of maxillary bones include their proximity to the oral immune barrier and frequent osteoclastogenesis caused by periodontal inflammation, dentoalveolar infection, and other oral lesions [24,39]. In all patients with MRONJ, the presence of BPs in the jaw bones was observed [23], and the BPs bound to the jawbone are thought to interact with the oral immune barrier [24]. BPs increase osteoclast apoptosis, resulting in decreased bone resorption and remodeling. Cell proliferation, adhesion, migration, and osteogenic differentiation of stem cells in the periodontal ligament are significantly decreased because of osteonecrosis [40,41]. BPs can induce the production of reactive oxygen species, which inhibit the proliferation and migration of oral fibroblasts [42]. Although BPs affect osteoclast function throughout the skeletal system, only the jaws can suffer from MRONJ—the mandible two times more often than the maxilla [33,34]. This may be attributed to a higher frequency of infection in the mandible due to low vascularity [43].

The data from our study corroborated the data extracted from the literature, showing us that treatment with BPs, in this case, zoledronic acid, has effects on all tissues of the oral cavity: bone, periosteum, oral mucosa, and immune cells.

The histopathological analysis in the present study showed that bone remodeling was affected, as all bone cell lines (osteoclasts, osteoblasts, and osteocytes) were influenced by MRONJ. The histopathological appearance of the bone showed the presence of multiple foci of necrosis, without inflammatory infiltration, with sharp, scalloped bone edges. Thus, the lack of osteocytes in the osteocyte lacunae, and the absence of osteoclasts and osteoblasts in histopathological preparations were observed [34]. On the histopathological images of the normal bone adjacent to the necrotic lesion, the presence of inflammatory infiltrate with many newly formed vessels was observed. Images from the periosteum showed an inflammatory reaction with multinucleated giant cells resulting from merging macrophages and fragments of necrotic bone tissue. Deep periodontium presents a defense reaction of the body around the area of necrosis, diffuse inflammatory infiltrate with immune cells of the neutrophil type, lymphocytes, and macrophages, which alter (dissect) the structure, chronic abundant inflammatory infiltrate of the lymphoplasmacytic type, numerous blood capillaries with turgescent endothelium. Aspects of histopathological sections support the etiopathogenic mechanisms described in animal studies. For each type of tissue lesion, there is a characteristic histopathological appearance [34]. Bone remodeling is a lifelong process that serves to adjust skeletal architecture and repair micro damage to maintain the functional integrity of bone. This process is characterized by the coupling of cells related to bone remodeling, osteoclasts for bone resorption, and osteoblasts for bone formation, which are organized into multicellular bone units [40]. Bisphosphonates and other antiresorptive drugs inhibit osteoclast differentiation and cause cell death. In addition, adequate bone remodeling capacity is thought to be critical in defense against infection and micro-fracture accumulation [43,44]. Increased bone resorption under oral conditions, coupled with the overlying thin mucosa and a direct route of bone contact with the external environment via the periodontal ligament, make the jaws a fertile ground for the development of MRONJ [45]. BPs can damage periodontal ligament stem cells by inducing apoptosis in a dose-dependent manner [46,47]. In the present study, we observed an absence of osteoclasts, as well as reversal lines. BPs inhibit osteoclast differentiation and function, increase osteoclast apoptosis, and ultimately lead to decreased bone remodeling and resorption [48,49,50,51,52]. These conditions can be affected by triggers such as dental surgery, including tooth extraction, microtrauma of the jaw, and local inflammation, leading to necrosis and exposure of the jawbone [53]. Since the bone turnover rate of alveolar bone is more than ten times faster in jaw bones than in long bones, it appears that the phenomenon is related to the ability to contain much more BPs in alveolar bone compared to bones elsewhere [54]. Although there are objections that bone turnover is not reduced in osteonecrosis lesions [55], and active bone resorption occurs due to the presence of osteoclasts in osteonecrotic areas [56,57], this hypothesis is further strengthened by the favorable results of teriparatide, which stimulates osteoclast activity [37,58,59]. Exploring the differences between myeloid lineage progenitor cell populations in the alveolar bone (mandibular) versus the long bone (femur), although there was no significant difference in progenitors, indicated that the population was significantly decreased in the mandibular bone marrow. T lymphocyte subsets were not significantly different between mandibular and femoral bone, except for CD4 regulatory T lymphocytes, which were significantly increased in the mandible, and B lymphocytes, which were also significantly increased in the mandible [60].

N-BPs (nitrogen bisphosphonates) interact with soft tissue cells such as fibroblasts and keratinocytes, producing gingival ulceration followed by bone exposure, the main clinical sign seen in BRONJ. Impaired soft tissue biological activity may result in delayed mucosal healing after tooth extraction or dentoalveolar surgery in patients treated with BPs [61]. It is assumed that the existence of inflammation in the jawbones (due to periodontitis, extractions, etc.) releases BPs from the bone, which causes inhibition of keratinocyte growth, leading to bone exposure, especially in patients with recent extractions [32]. Once bacteria invade the mucosal barrier, the host’s innate immune response is triggered, producing inflammation to restrain pathogens and maintain homeostasis [62].

Inflammation, a manifestation of the body against infection, is characterized by persistent infiltration of immune cells and increased levels of multiple pro-inflammatory cytokines and chemokines [63]. Inflammation was confirmed to be a pathological feature of MRONJ [64]. An increase in bacterial infiltration and inflammation at the necrotic site was observed in several previous studies [65]. BPs, on one hand, can induce immunosuppression by suppressing the activation of immune cells, and on the other hand, they can generate an imbalance between anti-inflammatory and pro-inflammatory cytokines, thus resulting in intense inflammation and tissue damage [32]. Histopathology and immunohistochemistry studies try to clarify the role played by various types of cells in MRONJ development, as they are also involved in the specific pathology of the oral cavity (reactions that occur in tissues due to periodontal disease, periapical lesions, and after tooth extractions). Currently, findings regarding the pathogenesis of MRONJ have been classified into several hypotheses, including altered bone remodeling, inflammation, or infection, altered immunity, soft tissue toxicity, and inhibition of angiogenesis [32].

The present study showed that the expression of immunohistochemical markers varied according to etiopathogenic and prognostic factors. The duration of drug administration is correlated with the intensity of immunohistochemical expression of all studied markers. The markers’ expression for patients from Craiova was generally higher for all markers compared to patients from Constanta, and the differences were statistically significant for two markers, αSMA, and mast cells. For 8 of the 9 markers (all except CD79α), there was a moderate or strong negative association between treatment duration and marker expression that was statistically significant. For an increasing trend of the duration of treatment, we identified in opposition a decreasing trend of markers’ scores. The markers’ expression according to the trigger factors varied from Low to Moderate-Strong. Overall, marker expression for periodontal disease-triggered MRONJ was higher (Moderate-High) for all markers, followed by lower values for extraction-triggered MRONJ (Low-Moderate) and low values for periapical infection-triggered MRONJ. Only for αSMA, expressions were statistically significantly different between different triggers. There are statistically significant differences in markers’ expression between periodontal disease and periapical infection. In a histopathological and immunohistochemical study of specimens from patients with periodontal disease, it was shown that periodontal disease is the result of bacterial aggression that triggers inflammation and mobilizes the immune system as a defense system. The inflammatory process observed as a local immune response, arising as a reaction to bacterial invasion, is characterized by the presence of defense cells, especially immune cells. Lymphocytes are present both in the superficial area of the chorion, immediately subepithelial, and in the rest of the chorion, having the appearance of a diffuse infiltrate or, in some cases, they were identified as a grouped, nodular, especially perivascular formation, indicating an increase in vascular permeability. Vascularization was increased, being located both subepithelial and in the rest of the chorion, due to angiogenic factors associated with mediators of the inflammatory process. The angiogenesis process was exclusively capillary, starting from pre-existing vessels [66]. Bănică and colleagues showed that macrophages appear frequently at the level of the periapical granuloma [67]. The density of macrophages explains the cellular and tissue disturbances that occur in the apical region of the tooth, under the influence of the bacterial flora that has arrived in this area, which has the role of phagocytizing pathogens, dead cells, and tissue residues resulting from bacterial aggression. Plasma cell reaction varied greatly, from apical granulomas with a moderate reaction, to granulomas with an intense plasma cell reaction. The plasma cell reaction was associated with the age of the granulomas, being more intense in the elderly, and in old, neglected granulomas compared with recent granulomas. Regardless of the age of the granuloma, the presence of a large number of mast cells was observed, especially around the blood vessels, as inhomogeneous cells, with a diffuse outline, due to the mast cell granulation processes. Although they are not directly involved in the body’s defense mechanisms, mast cells, via their mediators, contribute to the increase in the local blood flood and the accumulation of a greater number of immune cells. T and B lymphocytes had varied reactions from one case to another and even from one area to another in the same granuloma. In general, the two main types of lymphocytes had an average reaction [67].

In our study, markers’ expression for maxillary MRONJ patients was generally higher for all markers (mostly intense expression) compared to mandibular MRONJ patients (mostly low and moderate expression) and the differences were not statistically significant. Markers’ expression by lesion healing showed that expressions for healed patients were generally higher for all markers (mostly moderate expression) compared to relapsed patients (mostly low expression), and the differences were not statistically significant. For the subgroup of cured patients, the following markers were mostly moderately expressed: αSMA, T/CD8, B/CD20, PLASM/CD79α, and MASTOCYTES, while intense expression was predominant for T/CD3, T/CD4, C D68 and CD204. In contrast, the Relapse group emphasized more low expressions. For the subgroup of relapsed patients, only αSMA and B/CD20 were mostly expressed as moderate, while all others showed low intensities. An environment in which the immune system is suppressed favors the occurrence of MRONJ. N-BPs cause immune system dysfunction in MRONJ patients [68,69], affecting their ability to respond appropriately to immunological stress, independently of the oral microbiome [70]. It is important to note that a significant percentage of patients who develop MRONJ also have other conditions or are undergoing multiple pharmacological treatments, such as chemotherapy, steroid administration, antiviral drugs, etc. These conditions and treatments may contribute to weakening their immune system, negatively impacting immune system health [35,70].

Recently, the important role of immune responses and inflammation in the development and progression of BP-induced osteonecrosis of the jaw (MRONJ) was emphasized as a result of massive infiltration of lymphocytes mixed with inflammatory cells in the tissue affected by MRONJ [71]. BPs stimulate the production of mediators of acute inflammation in vitro [72] and in vivo [73], altering immune cell subpopulations [74,75], while markers of bone inflammation do not undergo changes. Healing of a tooth socket after extraction involves several immune processes. Initially, at the site of the injury, the T cell subpopulation releases cytokines, such as IL-17, which directly stimulates the proliferation and differentiation of local mesenchymal stem cells into bone cells. Then, a specific subset of T cells blocks the secretion of pro-inflammatory factors, thus facilitating the healing process of the injury. Under pathological conditions, excessive production of IL-17 exerts an adverse effect on bone cells, by inhibiting their differentiation and activity, as well as by promoting bone resorption by osteoclasts (OC) [76,77]. Therefore, a correct interaction between immune cells and bone cells is essential to prevent both bone and immune changes [78]. An animal model study reported that the administration of anti-inflammatory drugs or antibiotics significantly blocked zoledronic acid-induced osteonecrosis after tooth extraction [79], suggesting that this type of treatment should be considered in the prevention of MRONJ. Thus, the therapeutic approach using anti-inflammatory drugs or antibiotics shows the potential to block the development of BP-induced osteonecrosis after tooth extraction [79]. These findings indicate the need to explore this type of treatment for the prevention and management of MRONJ [80].

The Immune system is closely related to bone loss and bone regeneration. More recent data investigate the effects of antiresorptive drugs on components of the immune system and introduce potential changes in the immune response as novel elements in the pathogenesis of MRONJ [81]. The altered immune response of the host is another factor considered as important as the infection itself. Immune cells and macrophages are involved in the wound-healing process [82]. It has been suggested that macrophages may initially bind to BPs instead of osteoclasts, and the presence of BPs significantly alter macrophage viability and morphology in vitro [83]. This theory seems valid considering the lack of affinity between BPs and osteoclasts and the higher accumulation of these drugs in the jaws compared to the rest of the skeleton [16,84].

Gamma delta T cells representing innate lymphocytes are important in bone regeneration. Such T cells are significantly reduced in osteoporotic patients who are treated with BPs, indicating a link between MRONJ and gamma delta T cell deficiency [85]. Neutrophils promote healing after non-infectious injuries. N-BPs alter the defense capacity of neutrophils and impair normal wound healing, possibly representing a critical role in the pathogenesis of MRONJ [86]. To identify T lymphocytes in the present study, 3 markers were used: CD3, a marker that signifies the activity of the T lymphocyte population in general, CD4, a specific marker for the CD4+ helper T lymphocyte population, and CD8 marker, a specific marker for the CD8+ T lymphocyte population cytotoxic. TCD4+ helper lymphocytes contribute to the coordination of immune system responses by activating the appropriate effector mechanisms to eliminate the invading pathogen. More recently, other types of T helper lymphocytes were described, especially Th17 and regulatory T cells (Treg) [87]. Th17 cells promote defense against extracellular bacterial and fungal infections and play an important role in maintaining the integrity of mucosal barriers. Treg cells suppress or “regulate” other immune cells. In this way, Treg cells help limit the acute or chronic inflammatory response, as well as harmful responses to self-antigens (autoimmune responses). Differentiated T cell populations are resident in healthy oral tissues, including the gingiva, buccal mucosa, tongue, and sublingual regions. This includes CD8+ and CD4+ T cells that are Th1, Th17, or Treg cells. Most of these T cells show a resident memory phenotype and are therefore ready to respond if the local oral barrier is breached in any way [88].

Macrophages are other immune cells sensitive to BPs, which have an inhibitory effect and reduce the viability and differentiation capacity of macrophages. Macrophage function is disrupted by increased matrix metalloproteinases (MMP) expression, leading to reduced wound healing in areas affected by MRONJ [37,89]. In vitro studies suggest that BPs disrupt the local immune function of macrophages, directly affecting their survival, migration, differentiation, and phagocytic activity. On the other hand, in vivo, clinical, or translational studies clearly capture an altered macrophage function in MRONJ given by BPs or associated RANKL inhibitors [81]. Changes in macrophage polarization and function are a response to a sustained inflammatory environment that propagates the extent and severity of MRONJ [81]. There is a direct connection between osteoclasts and macrophages. In the jaws, osteoclasts remove necrotic bone in the inflammatory environment during periodontal disease, or in sockets after extraction. Given the close association of the jaws with the oral or sinus mucosa, osteoclast function is even more important in the oral environment, where pharmacologically mediated defective osteoclast function alters oral wound healing and plays a key role in macrophage failure. Failure of necrotic bone removal and normal oral bone, and oral mucosa healing alters macrophage function, cytokine secretion, and polarization and propagates a pro-inflammatory environment, in a positive feedback mechanism that ultimately leads to the development, progression, and expansion of MRONJ [81]. Macrophages, due to their primary involvement in the elimination of pathogens, cytotoxic molecules, and dead cells, have developed a repertoire of diverse scavenger receptors (SRs) with the ability to detect a broad spectrum of ligands. CD68 is also considered a member of the SR family as a scavenger receptor type D (SCARD) because it can be significantly upregulated in macrophages responding to inflammatory stimuli [32].

Inhibition of angiogenesis is another central hypothesis believed to be a possible cause of pathogenesis of MRONJ. Inhibition of angiogenesis is hypothesized to adversely affect bone regeneration capacity after bone injury, delay bone remodeling or healing, and may increase susceptibility to superinfection. Zoledronate has direct inhibitory effects on angiogenesis, with MRONJ occurring due to reduced angiogenesis affecting healing after surgery [37,90]. Bisphosphonates are thought to inhibit bone angiogenesis by suppressing the growth of vascular endothelial cells, leading to avascular necrosis of the bone. There are several reports showing that BPs directly inhibit angiogenesis in vitro or in vivo [91,92,93], although studies on the effects of BPs on angiogenesis in the bone marrow and periosteum need to be carried out. BPs may exert indirect angiogenesis-suppressive effects in the bone, as osteoclasts are required for the passage of new vessels into the bone matrix [82,94].

Another studied marker was αSMA. Actin alpha 2 (ACTA2), also known as alpha-smooth muscle actin (αSMA), is one of six actin isoforms that form the cytoskeleton and is found predominantly in smooth muscle cells. ACTA2 plays a crucial role in cells, promoting focal adhesion, migration, transcriptional and shape regulation, and contractile activity. In the oral cavity, ACTA2 is expressed in periodontal tissues and supports cytoskeletal dynamics, cell motility, and contractility [95]. Vascular endothelial growth may be a critical factor in the pathogenesis of MRONJ [96]. Since postmenopausal women have a higher risk of periodontal disease due to the decrease in estrogen levels, which exerts a trophic action on the oral cavity [97], this may explain how the oral microcirculatory alterations observed in postmenopause can amplify the adverse effects of some drugs, such as zoledronic acid, on oral health [97].

The commensal microbiome may play a protective rather than a pathological role in the early stages of MRONJ development [98]. Invasive dental treatments (IDT) and periodontal disease (PD) were considered potential risk factors for MRONJ; however, the association between these exposures and MRONJ remains controversial [99]. Dental treatments are considered invasive when they cause bleeding and introduce oral bacteria into the bloodstream, as in extractions, scaling and root planning, implant placement, and any type of oral surgery. They can produce temporary bacteremia capable of causing microbial immune subversion that triggers systemic inflammation. The directed histological evaluation shows the high incidence of Actinomyces infection in drug-related osteonecrosis of the jaw [100], which was also observed in our study [35]. The critical role of bacterial infection in the pathogenesis of MRONJ can be justified by the decreased incidence in patients, after improved dental hygiene [100,101,102,103]. Bisphosphonate-loaded bone is more susceptible to infections, not only because of the suppression of defense mechanisms, especially osteoclast activity and bone remodeling but also because the bisphosphonate-loaded bone is more prone to bacterial colonization [31].

A limitation of this study is the absence of data from cancer patients without MRONJ but under treatment with zoledronic acid—to compare the effects of zoledronic acid on MRONJ patients with non-MRONJ patients (nonethical, since surgical interventions are carried out for MRONJ, and only in extractions cases a comparison could be carried out—that was not the case). Follow-up work includes a new study lot characterized by other inclusion criteria.

## 4. Materials and Methods

### 4.1. Study Design

The retrospective study used databases from Dolj County Emergency Clinical Hospital and Constanța County Emergency Clinical Hospital, as well as from the Center for Microscopic Morphology and Immunology in Craiova, Romania. The database contains information on patient demographics (age, sex), cancer diagnosis, comorbidities, MRONJ, surgical treatment of MRONJ, and histological and immunohistochemical data. The study data were collected between March 2019 and December 2022. The Ethics Committee of the University of Medicine and Pharmacy in Craiova approved the study with no. 59/22.03.2019. The study meets the STROBE criteria [103].

The primary outcome was to correlate the immune response defined by the expression of immunohistochemical markers (αSMA, CD3, CD4, CD8, CD20, CD79α, CD68, CD204, tryptase) with etiopathogenic factors of MRONJ, by using the information obtained from the immunohistochemical analysis of MRONJ tissues from cancer patients treated with zoledronic acid. Secondary outcome referred to establishing an overall marker score for each patient, and its relationship with the trigger factors.

### 4.2. Patients

The retrospective study included a group of 51 patients with cancer under bisphosphonate treatment for bone metastasis who suffered complications of osteonecrosis of the jaw and presented themselves for treatment at the Department of Oral and Maxillo-Facial Surgery of the Craiova County Clinical Hospital or the Oral and Maxillo-Facial Surgery department of the Constanta County Clinical Hospital in the period March 2019–December 2022. All participating patients gave their consent for the treatment and the histological and immunohistochemical processing of the obtained specimens.

#### 4.2.1. Criteria for Inclusion in the Study

All patients included in the study were patients with various types of cancer, treated for bone metastases with bisphosphonates, who developed osteonecrosis of the jaw bones during treatment, such as exposed necrotic bone or intra- or extraoral fistulas with persistence greater than 8 weeks, without previous radiation therapy in the jawbones area, and without cervicofacial cancers or metastases.

#### 4.2.2. Exclusion Criteria in the Study

Patients with malignant tumors in the maxillary area and patients who received radiation therapy in the facial area in the past were excluded from the study.

### 4.3. Surgical Intervention

From the data extracted from the medical charts, each patient received the indication of local decontamination of the oral cavity with 0.2% chlorhexidine mouthwash twice a day and topical application on the lesion with 1% chlorhexidine gel three times a day, as well as empiric oral antibiotic therapy with amoxicillin and clavulanic acid 875/125 mg twice daily for non-beta-lactam allergic patients and clindamycin 600 mg twice daily for allergic patients. Before the institution of empiric antibiotic therapy, the antibiogram was collected from the lesion. Inpatients were subsequently switched to targeted IV antibiotic therapy according to the antibiogram result.

The surgical interventions were performed with general or local anesthesia, with an intraoral approach, and consisted of curettage and sequestrectomy in most cases, with beveling of the sharp edges of the bone and closing the wound with a mucoperiosteal flap. In cases of recurrence, a second surgical intervention included bone resection associated with osteosynthesis (when appropriate) and reconstruction with proximity flaps. In all cases, the removal of bone sequestrations and curettage into the bone tissue was performed until clear bleeding from the underlying bone occurred. A-PRF has been used in very few cases, especially when the resulting bone defect could not be covered with a mucoperiosteal flap or when it was difficult to protect the remaining bone. Due to the long half-life of zoledronic acid, the surgeon did not recommend discontinuation of zoledronic acid. Treatment with zoledronic acid was stopped only in patients who received this recommendation from their primary care oncologists.

After surgery, antibiotic therapy continued for 10 to 14 days, intravenously, until suture removal (in severe cases), followed by oral antibiotic therapy for 7 days. In less severe cases, after discharge, the patient continued oral antibiotic therapy until 2–3 weeks after the surgical procedure.

Age, sex, type of cancer, type of bisphosphonate, location of osteonecrosis, and stage of osteonecrosis were recorded for each patient.

### 4.4. Histological and Immunohistochemical Evaluation

In the treatment of osteonecrosis, patients underwent sequestrectomy or partial resection of the necrotic bone. The bone and marginal periodontal specimens taken at the Oral and Maxillofacial Surgery Clinics (Dolj County Clinical Hospital, Constanța County Clinical Hospital) were included in 10% formalin (*v*/*v*) and sent to the Department of Histopathology where they were processed for histopathological analysis and immunohistochemistry. The histological and immunohistochemical analysis was carried out at the Research Center for Microscopic Morphology and Immunology Studies, University of Medicine and Pharmacy of Craiova. A total of 78 samples were collected.

Immunohistochemical evaluation of bone changes in bisphosphonate-induced osteonecrosis in cancer patients treated with IV zoledronic acid who developed osteonecrosis and underwent partial resection/sequestrectomy of necrotic bone as part of osteonecrosis treatment included a histopathological study and an immunohistochemical study.

The histopathological study followed the highlighting of the main tissue changes in osteonecrosis to zoledronic acid and their inclusion in the main pathogenic mechanisms of MRONJ. Of the total of 78 samples, 16 were damaged during the fixation and staining procedures, resulting in a final number of 62 samples included in the study. The samples were stained using the hematoxylin-eosin (HE) and the trichrome technique, according to the Goldner–Szekely (GS) method. A qualitative histopathological analysis was performed, taking into account changes in the epithelial, periosteal, deep periodontal, and alveolar bone levels. The analysis was performed on samples collected from osteonecrosis foci located in the anterior and posterior areas of the upper and lower jaws. These samples were used to analyze the following components: inflammatory infiltration, blood vessels, osteocytes and empty lacunae, and viable bone. Analysis of histological parameters was performed by a single calibrated investigator using a Nikon Eclipse 55i binocular microscope equipped with planar-fluorine objectives, DS-Fi1(5Mp) digital camera as well as Nikon NIS-Elements image acquisition and analysis software (Nikon, Tokyo, Japan). Images were captured using 100× and 200× objectives. The clinical diagnosis of osteonecrosis of the jaw bones was confirmed by the results of the histopathological examination, the general histopathological evaluation revealing different degrees of osteonecrosis.

The immunohistochemical study of the oral tissues (oral mucosa, periodontium, bone, periosteum) from the area of osteonecrosis to zoledronic acid sought to highlight the main types of cells involved in the immune response and their role in the pathogenetic mechanisms of osteonecrosis. The studied cells were T lymphocytes, B lymphocytes, plasma cells, macrophages, mast cells, keratinocytes, and endothelial cells; and the immune response of patients with cancer and MRONJ to zoledronic acid was highlighted depending on the duration of treatment with bisphosphonates, the location of MRONJ, the trigger factor (periodontal disease, extraction, periapical pathology), and wound healing after surgery. The following antibodies were used: AE1/AE3, monoclonal antibodies used to detect cytokeratins, which are the predominant structural proteins in epithelial cells; alpha smooth muscle Actin or αSMA (ACTA2) Mouse Mono-clonal Antibody, an antibody for identification of myofibroblasts and smooth muscle cells in vascular walls; CD3 (mouse monoclonal anti-human CD3, clone F7.2.38, Dako, dilution 1:25) for enhancing T lymphocytes; CD4, for highlighting CD4 positive T lymphocytes, clone MT310, M0716, Dako, dilution 1:50; CD8, for highlighting CD8 positive T lymphocytes, clone C8/144B; CD20 (mouse monoclonal anti-human CD20cy, clone L26, Dako, dilution 1:50) for B-lymphocyte study; CD79alpha (mouse anti-human monoclonal CD79-alpha, clone JCB117, Dako, dilution 1:50) for plasma cells; CD68 (mouse anti-human monoclonal CD68, clone KP1, Dako, dilution 1:200) for macrophages; tryptase (monoclonal mouse anti-human mast cell tryptase, clone AA1, Dako, dilution 1:500) for mast cell study.

The qualitative assessment of the immunohistochemical reaction to the studied markers was achieved by assessing the intensity of the staining as follows: the intensely positive reaction (+++) was defined as the reaction present in more than 80% of the cells, diffusely distributed, well visible under microscopic examination with low magnification objectives; the reaction of moderate intensity (++) was defined as the reaction present in 30–80% of the cells, with a focal disposition, well visible during microscopic examination with objectives with medium power of magnification; the reaction of weak intensity (+) was defined as the reaction present in 5–30% of the cells, visible during microscopic examination with high-power objectives; and the reaction was absent (−) when no cell with positive immunomarking can be identified.

Immunohistochemical analysis was performed by a single calibrated investigator using a Nikon Eclipse 90i binocular microscope (Nikon, Tokyo, Japan) equipped with planar-fluorine objectives, DS-Fi1(5Mp) digital camera, and Nikon NIS image acquisition and analysis software -Elements (Nikon, Tokyo, Japan). The images were captured using 100× and 200× objectives.

### 4.5. Statistical Analysis

All primary data acquired in this study were recorded using Microsoft Excel 365 (San Francisco, CA, USA), and included clinical and non-clinical data related to demographic aspects (gender, age, medical center), primary tumors’ location and comorbidities, BP treatment (acid type and duration), MRONJ data (trigger factor, duration, current stage, location, relapse status), and immunohistochemical markers’ expression. Continuous variables were expressed as mean ± standard deviation (SD) to complete the descriptive analysis, while categorical data were expressed as absolute and relative frequencies (%). All statistical analyses were performed using IBM SPSS Statistics 20.0 software (IBM Corp., New York, NY, USA). Potential associations involving the acquired parameters were analyzed using the following tests: Shapiro–Wilk’s for normality distribution, Levene’s test for homogeneity of variances, point-biserial or Kendall’s tau-b for correlations, Mann–Whitney U, and Kruskal–Wallis H (completed with pairwise comparisons performed using Dunn’s procedure with a Bonferroni correction for multiple comparisons) for group distributions. Chi-square and Fisher Exact tests (χ^2^) were employed for categorical parameters. The following p values were accepted: *p* < 0.05 significant based on a confidence interval (CI) of 95%, as well as *p* < 0.001 highly significant (CI of 99.9%).

## 5. Conclusions

The immunohistochemical expression of the markers is statistically significantly lower with increasing duration of bisphosphonate treatment. The patient’s immune response was negatively influenced by the duration of the treatment, the trigger factor, the location of the lesion in the mandible, and the recurrence.

## Figures and Tables

**Table 1:** Distribution of the study group according to healing status, demographic, and clinical data.

Parameter	Value	N (%)			*p*
		Overall (100%)	Healed (100%)	Relapse (100%)	
Gender	Females	32 (62.7)	24 (75.0)	8 (25.0)	0.584 *
	Males	19 (37.3)	14 (73.7)	5 (26.3)	
Age	mean ± SD	69.94 ± 8.50	68.39 ± 7.93	74.46 ± 8.79	0.025 **
Medical center	Craiova	26 (51.0)	21 (80.0)	5 (19.2)	0.296
	Constanta	25 (49.0)	17 (68.0)	8 (32.0)	
Trigger factor	Periodontal disease	7 (13.7)	5 (71.4)	2 (28.6)	0.900
	Extraction	34 (66.7)	26 (76.5)	8 (23.5)	
	Periapical disease	10 (19.6)	7 (70.0)	3 (30.0)	
Treatment duration	mean ± SD	29.51 ± 9.50	28.26 ± 8.82	33.15 ± 10.78	0.110 **
	12–35 (months)	30 (58.8)	25 (65.8)	5 (38.5)	0.084 ***
	>36 (months)	21 (41.2)	13 (34.2)	8 (61.5)	
MRONJ stage	2	39 (77.6)	33 (86.8)	5 (13.2)	0.002 *
	3	12 (22.4)	4 (36.4)	7 (63.6)	
MRONJ localization	Lower jaw	34 (66.7)	25 (73.5)	9 (26.5)	0.553 *
	Upper jaw	17 (33.3)	13 (76.5)	4 (23.5)	
MRONJ localization in the jaw	Posterior	47 (92.2)	34 (72.3)	13 (27.7)	0.295 *
	Anterior	4 (7.8)	4 (100)	0 (0)	
HTA	No	19 (37.3)	14 (73.7)	5 (26.3)	0.584 *
	Yes	32 (62.7)	24 (75.0)	8 (25.0)	
Obesity	No	37 (72.5)	28 (75.7)	9 (24.3)	0.508 *
	Yes	14 (27.5)	10 (71.4)	4 (30.8)	
Hormonotherapy	No	39 (76.5)	31 (79.5)	8 (20.5)	0.138 *
	Yes	12 (23.5)	7 (58.3)	5 (41.7)	

* Fisher Exact test. ** Point-biserial text. *** Chi-Square test.

**Table 2:** Distribution of the study group according to healing status and histological data.

Parameter	Value	N (%)			*p*
		Overall	Healed	Relapse	
Bacterial colonies	No	24 (47.1)	17 (70.8)	7 (29.2)	0.570
	Yes	27 (52.9)	21 (77.8)	6 (22.2)	
Inflammatory infiltrate	No	4 (7.8)	3 (75.0)	1 (25.0)	0.734 *
	Yes	47 (92.2)	35 (74.5)	12 (25.5)	
Lymphoplasmacytic infiltrate	No	13 (25.5)	10 (76.9)	3 (23.1)	0.566 *
	Yes	38 (74.5)	28 (73.7)	10 (26.3)	
Viable bone	No	35 (68.6)	27 (77.1)	8 (22.9)	0.378 *
	Yes	16 (31.4)	11 (68.8)	5 (31.2)	

* Fisher Exact.

**Table 3:** Markers’ expression for immunohistochemical analysis in the study group.

	αSMA	LyT/CD3	LyTh/CD4	LyTc/CD8	LyB/CD20	Plasmocites/CD79	Macrophages/CD68	Macrophages/CD204	Mastocytes/Tryptase
	N (%)	N (%)	N (%)	N (%)	N (%)	N (%)	N (%)	N (%)	N (%)
Negative	4 (7.84)	6 (11.76)	8 (15.69)	7 (13.73)	11 (21.57)	14 (27.45)	6 (11.76)	9 (17.65)	11 (21.57)
Low	8 (15.69)	13 (25.49)	14 (27.45)	13 (25.49)	13 (25.49)	16 (31.37)	12 (23.53)	13 (25.49)	18 (35.29)
Moderate	30 (58.82)	16 (31.37)	15 (29.41)	17 (33.33)	21 (41.18)	19 (37.25)	16 (31.37)	15 (29.41)	20 (39.22)
Intense	9 (17.65)	16 (31.37)	14 (27.45)	14 (27.45)	6 (11.76)	2 (3.92)	17 (33.33)	14 (27.45)	2 (3.92)
Total	51 (100)	51 (100)	51 (100)	51 (100)	51 (100)	51 (100)	51 (100)	51 (100)	51 (100)

**Table 4:** The relationship between BP treatment duration and IHC markers’ expression.

Marker	Correlation Coefficient	*p* *
αSMA	−0.234	0.048
CD3	−0.340	0.003
CD4	−0.418	<0.0005
CD8	−0.418	<0.0005
CD20	−0.292	0.015
CD68	−0.291	0.012
CD79	−0.188	0.109
CD204	−0.420	<0.0005
MASTOCITE	−0.248	0.035

* Kendall’s tau-b correlation.

**Table 5:** Distribution of patients according to the trigger factor and markers’ expression.

Marker	Extraction N (%)				Periodontal Disease N (%)				Periapical Infection N (%)				*p*
	N *	L *	M *	I *	N *	L *	M *	I *	N *	L *	M *	I *	
αSMA	4 (11.76)	4 (11.76)	20 (58.82)	6 (17.65)	0 (0)	0 (0)	4 (57.14)	3 (42.86)	0 (0)	4 (40)	6 (60)	0 (0)	0.048 *
CD3	5 (14.71)	9 (26.47)	9 (26.47)	11 (32.35)	0 (0)	1 (14.29)	3 (42.86)	3 (42.86)	1 (10)	3 (30)	4 (40)	2 (20)	0.439
CD4	6 (17.65)	10 (29.41)	8 (23.53)	10 (29.41)	0 (0)	1 (14.29)	3 (42.86)	3 (42.86)	2 (20)	3 (30)	4 (40)	1 (10)	0.214
CD8	6 (17.65)	9 (26.47)	9 (26.47)	10 (29.41)	0 (0)	1 (14.29)	3 (42.86)	3 (42.86)	1 (10)	3 (30)	5 (50)	1 (10)	0.311
CD20	10 (29.41)	5 (14.71)	13 (38.24)	6 (17.65)	0 (0)	2 (28.57)	5 (71.43)	0 (0)	1 (10)	6 (60)	3 (30)	0 (0)	0.480
CD68	5 (14.71)	8 (23.53)	9 (26.47)	12 (35.29)	0 (0)	1 (14.29)	3 (42.86)	3 (42.86)	1 (10)	3 (30)	4 (40)	2 (20)	0.484
CD79	11 (32.35)	10 (29.41)	11 (32.35)	2 (5.88)	1 (14.29)	1 (14.29)	5 (71.43)	0 (0)	2 (20)	5 (50)	3 (30)	0 (0)	0.382
CD204	7 (20.59)	9 (26.47)	8 (23.53)	10 (29.41)	0 (0)	1 (14.29)	3 (42.86)	3 (42.86)	2 (20)	3 (30)	4 (40)	1 (10)	0.220
Tryptase	7 (20.59)	13 (38.24)	13 (38.24)	1 (2.94)	0 (0)	3 (42.86)	4 (57.14)	0 (0)	4 (40)	2 (20)	3 (30)	1 (10)	0.499

* N—Negative, L—Low, M—Moderate, I—Intense.

**Table 6:** Markers expression for immunohistochemical analysis in the study group according to MRONJ localization (upper or lower jaw).

Marker	Upper Jaw N (%)				Lower Jaw N (%)				*p*
	N *	L *	M *	I *	N *	L *	M *	I *	
αSMA	2 (11.76)	2 (11.76)	3 (17.65)	8 (47.06)	2 (5.88)	5 (14.71)	22 (64.71)	5 (14.71)	>0.05
CD3	3 (17.65)	3 (17.65)	2 (11.76)	5 (29.41)	3 (8.82)	11 (32.35)	11 (32.35)	9 (26.47)	>0.05
CD4	5 (29.41)	5 (29.41)	2 (11.76)	4 (23.53)	3 (8.82)	12 (35.29)	11 (32.35)	8 (23.53)	>0.05
CD8	4 (23.53)	4 (23.53)	2 (11.76)	5 (29.41)	3 (8.82)	11 (32.35)	12 (35.29)	8 (23.53)	>0.05
CD20	2 (11.76)	2 (11.76)	5 (29.41)	8 (47.06)	9 (26.47)	8 (23.53)	13 (38.24)	4 (11.76)	>0.05
CD68	3 (17.65)	3 (17.65)	2 (11.76)	5 (29.41)	3 (8.82)	10 (29.41)	11 (32.35)	10 (29.41)	>0.05
CD79	3 (17.65)	3 (17.65)	5 (29.41)	8 (47.06)	11 (32.35)	11 (32.35)	11 (32.35)	1 (2.94)	>0.05
CD204	5 (29.41)	5 (29.41)	2 (11.76)	4 (23.53)	4 (11.76)	11 (32.35)	11 (32.35)	8 (23.53)	>0.05
Tryptase	4 (5.63)	4 (5.63)	5 (7.04)	7 (9.86)	7 (20.59)	13 (38.24)	13 (38.24)	1 (2.94)	>0.05

* N—Negative, L—Low, M—Moderate, I—Intense.

**Table 7:** Markers expression for immunohistochemical analysis in the study group according to outcome (healed vs. relapsed).

Marker	Healed N (%)				Relapse N (%)				*p*
	N *	L *	M *	I *	N *	L *	M*	I *	
αSMA	3 (7.89)	5 (13.16)	22 (57.89)	8 (21.05)	1 (7.69)	3 (23.08)	8 (61.54)	1 (7.69)	>0.05
CD3	5 (13.16)	8 (21.05)	11 (28.95)	14 (36.84)	1 (7.69)	5 (38.46)	5 (38.46)	2 (15.38)	>0.05
CD4	6 (15.79)	9 (23.68)	11 (28.95)	12 (31.58)	2 (15.38)	5 (38.46)	4 (30.77)	2 (15.38)	>0.05
CD8	5 (13.16)	8 (21.05)	13 (34.21)	12 (31.58)	2 (15.38)	5 (38.46)	4 (30.77)	2 (15.38)	>0.05
CD20	8 (21.05)	10 (26.32)	14 (36.84)	6 (15.79)	3 (23.08)	3 (23.08)	7 (53.85)	0 (0)	>0.05
CD68	5 (13.16)	7 (18.42)	11 (28.95)	15 (39.47)	1 (7.69)	5 (38.46)	5 (38.46)	2 (15.38)	>0.05
CD79	10 (26.32)	12 (31.58)	15 (39.47)	1 (2.63)	4 (30.77)	4 (30.77)	4 (30.77)	1 (7.69)	>0.05
CD204	7 (18.42)	8 (21.05)	11 (28.95)	12 (31.58)	2 (15.38)	5 (38.46)	4 (30.77)	2 (15.38)	>0.05
Tryptase	8 (21.05)	11 (28.95)	17 (44.74)	2 (5.26)	3 (23.08)	7 (53.85)	3 (23.08)	0 (0)	>0.05

* N—Negative, L—Low, M—Moderate, I—Intense.

## Data Availability

The authors declare that the data of this research are available from the corresponding authors upon reasonable request.
